# Recurrence of perforation and overall patient survival after penetrating keratoplasty versus amniotic membrane transplantation in corneal perforation

**DOI:** 10.1007/s00417-022-05914-0

**Published:** 2023-01-21

**Authors:** Carolin Elhardt, Romina Schweikert, Rupert Kamnig, Efstathios Vounotrypidis, Armin Wolf, Christian M. Wertheimer

**Affiliations:** Department of Ophthalmology, Ulm University, Prittwitzstrasse 43, 89075 Ulm, Germany

**Keywords:** Corneal perforation, Corneal ulcer, Amniotic membrane, Perforating Keratoplasty

## Abstract

**Purpose:**

The following is a comparative analysis on the treatment outcomes of corneal perforations using amniotic membrane transplantation (AMT) or penetrating keratoplasty (PK).

**Methods:**

This monocentric retrospective study was performed at the Department of Ophthalmology, University Hospital Ulm, Germany. A total of 78 eyes of 78 patients were included. Thirty-nine eyes received an AMT, and 39 patients were treated with a PK. Primary outcome was recurrence of perforation. Secondary outcomes were patient mortality and visual acuity.

**Results:**

No statistically significant difference was observed with regard to a recurrence of perforation between the two groups (26% in AMT vs 23% in PK, *p* > 0.99). The time of recurrences was within the first two years and did not differ statistically (*p* = 0.97). In addition, a proportional hazards model with cox regression regarding recurrent perforation showed no significant differences (*p* = 0.5). After AMT, 41% and after KP, 28% of the patients died during follow-up (*p* = 0.2), respectively. The Charlson Comorbidity Index (*p* < 0.0001) and the age at the time of surgery (*p* = 0.0002) were statistically significantly higher in those who were deceased. A mean follow-up of 485 ± 517 days was recorded.

**Conclusion:**

Both surgical methods show good results and no statistically significant difference regarding recurrent perforation rate. About a third of the patients died during the follow-up period. The decision regarding the appropriate method should therefore be based on a combination of all factors.

**Supplementary Information:**

The online version contains supplementary material available at 10.1007/s00417-022-05914-0.


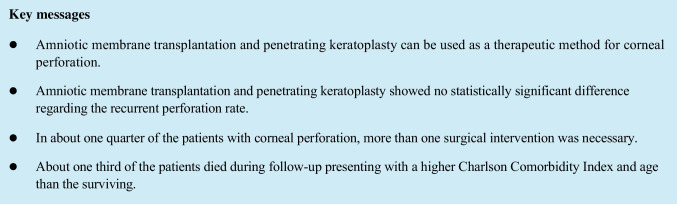


## Introduction

Corneal perforations are rated among the most serious conditions of the cornea with a reduced prognosis for visual restoration [1]. Corneal keratolysis and perforation can be caused by various pathologies, including trauma, infection, autoimmune diseases, ocular surface diseases, and other noninfectious causes [2]. Timely and accurate treatment is crucial to prevent further complications such as secondary glaucoma, choroidal hemorrhage, and endophthalmitis [2]. Often multistage surgical procedures are required to prevent these complications [3]. The primary goal is tectonic closure of the perforated area to restore the integrity of the eye. Subsequently, visual acuity can be addressed by further surgeries [4].

Penetrating keratoplasty (PK) is an option for the treatment of corneal perforation [5]. In addition, amniotic membrane transplantation (AMT) has also been used as a patch graft material to close the defect and replace affected corneal tissue [6]. It promotes corneal epithelialization and has anti-fibrotic, anti-inflammatory, anti-angiogenic, and antimicrobial properties [7, 8]. The choice of surgical method depends on the size, localization, and underlying cause of the corneal perforation, as well as the surgeon’s experience [4].

In literature, long-term tectonic success rates for both procedures are comparable, with a tendency in favor of PK, but to our knowledge no direct comparison has been performed. This retrospective study was designed to compare both methods under real-life conditions in terms of recurrence of perforation following the initial surgical procedure as a primary endpoint. As secondary endpoints, visual acuity and – due to a high loss of follow-up – comorbidities and mortality were analyzed.

## Materials and methods

### Study design

This is a retrospective controlled study of 78 eyes of 78 patients with corneal perforation due to different pathologies. After a sample size calculation, two groups were established recruiting patients back in time until a size of 39 patients was reached in both groups. All patients underwent one of two different types of surgery, either AMT (39 eyes) or PK (39 eyes) at the department of ophthalmology at University Hospital Ulm from 11/2015 to 09/2021. The retrospective analysis was approved by the ethics committee of Ulm University (ethical approval id: 178/21) and adhered to the Declaration of Helsinki.

### Inclusion and exclusion criteria

All patients were diagnosed with corneal perforation during the above-indicated period after a complete ophthalmological examination. Exclusion criterion was a follow-up of less than 30 days.

### Baseline examinations

The data analyzed included demographic information, medical history, complete slit lamp examination, postoperative best visual acuity, and overall survival. The size of corneal perforation was measured using ImageJ 1.80 (National Institutes of Health, Bethesda, MD, USA) [9]. To take the different image sizes into account, the size of the perforation is expressed as a percentage of the whole corneal size by measuring both the corneal defect and the entire cornea as a reference area in pixels.

### Surgical procedure

Both procedures for the preparation of amniotic membranes and corneal transplants adhered to the currently valid German guidelines. For AMT, cryopreserved fresh amniotic membranes were obtained from a government-licensed manufacturer for pharmaceutical production and stored at − 80 °C for a maximum of one year. In the AMT group, 11 patients underwent surgery under topical, 11 patients under local, and 17 patients under general anesthesia. After careful draping, the sizes of the corneal radius and of the corneal defect were measured with a caliper. The amniotic membrane was cut into compatible sizes. The defect was covered with the amniotic membrane as an inlay with epithelium up plus an overlay with epithelium down which was fixated with 10.0 nylon sutures in the cornea. If necessary, paracentesis was performed, e.g., to remove an iris tamponade, and a normal eye pressure was established. At the end of procedure, a bandage contact lens was applied in all cases.

For PK, corneal transplants from the eye bank of the Institute for Transfusion Medicine (University Hospital Ulm, Ulm, Germany) were used. They were cultivated according to German guidelines and with a government license for pharmaceutical production in warm environment (37 °C) in minimum essential medium (MEM) and 2% fetal calf serum (FCS) for a maximum of 34 days. All PKs were performed under general anesthesia. After careful draping, the corneal center was determined with a caliper and the eight cardinal suture positions were marked. The lenticel of the donor cornea was manually trephined and bedded on viscoelastic substance. The recipient’s cornea was trephined and excised. The donor cornea was first fixed with eight cardinal 10.0 nylon sutures and then with additional sutures on the recipient’s corneal margins, resulting in a total of 24 or 32 sutures, depending on the size of the donor corneal button.

### Follow-up

Accompanying the procedure, all patients received intensive topical treatment including steroids, antibiotic eyedrops, and moisturizing eye drops adapted to the cause of corneal perforation and the patients’ needs. Additionally, systemic steroids, antivirals, and antibiotics, as well as contact lenses were used. If necessary, a systemic workup was performed in collaboration with other specialties. Regular follow-up examinations at adequate intervals were performed in the outpatient clinic. In case of worsening or recurrent perforation, patients were readmitted to the inpatient ward and, if necessary, operated on again. The postoperative survival rate of patients was also evaluated in the study (bereavement records, documentation also from other departments from our clinic).

### Study endpoints

The primary endpoint of the study was the recurrence of perforation after the initial surgical procedure requiring further surgery. If no further surgeries were necessary for tectonic support, e.g., a second surgery was performed for visual rehabilitation only, the first surgery was defined as successful. Because the death of patients acts as a competing factor for the endpoint of recurrent perforation, patient death was additionally included in a hazard ratio model and mortality was calculated in both groups. To quantify the patients’ underlying medical conditions and risk of death, the Charlson Comorbidity Index [10] was calculated at the time of surgery from anesthesia protocols using the updated version of the score with 12 comorbidities [11]. Corrected visual acuity was measured at first presentation and at the last follow-up visit and converted from decimal values to logMAR.

### Statistical analysis

The primary endpoint of the study was the recurrence of corneal perforation to provide an indication of the success of the surgeries. In order to make a valid statement about the therapeutic success despite the high mortality rates in the patient groups, death was included in the analysis as a competing risk, since it prevents the recurrence of perforation. “Ordinary” censoring, on the other hand, does not affect the event of interest (recurrence of perforation) because neither the target “recurrence of perforation” nor an intercurrent event such as death occurred and the recurrence of perforation may continue unchanged, albeit unobserved. Therefore, the event of death must be treated differently from “ordinary” censoring, and after primary surgical treatment, a distinction was made between recurrence of perforation, death, or censoring. The cumulative hazards determined using Nelson-Aalen estimators describe the cumulative risk for an event (in this case, recurrence of perforation) occurring over time among the patients still at risk at that time who have not yet had an event and who have not been censored. The slope of the graph of the cumulative hazards gives the estimated hazards, which can be interpreted as the instantaneous risk for the event of a recurrent perforation (given that one has not yet had an event and has not yet been censored).

Excel 365 (Microsoft, Redmond, WA, USA) and SPSS 27 (IBM, Armonk, NY, USA) were used for data processing and statistical analysis. Statistical comparisons were performed using either Fisher’s exact test or Mann–Whitney *U*-test. A *p*-value of < 0.05 was considered significant. The Nelson-Aalen estimator was used to calculate the cumulative hazards for recurrent perforation and patient death, and a Cox proportional hazards model was calculated for both using R (V 4.1.2., R Core Team, Auckland New Zealand). Graphs were plotted in R and GraphPad Prism 9 (GraphPad Software, San Diego, CA, USA).

## Results

### Baseline characteristics

In each group, 39 eyes of 39 patients underwent either AMT or PK to restore tectonic integrity of the globe after corneal perforation due to 17 different pathologies. The two groups did not differ statistically significantly in age, gender distribution, laterality of eye, and follow-up time (*p* > 0.05). The size of the perforation, measured as a percentage of the whole corneal area, was slightly but significantly larger in patients who received PK (1.2% ± 0.7%) than in those who received AMT (0.8% ± 1.0%; *p* = 0.009). Endothelial cell count was available for 34 of the corneal grafts. In 4 grafts, the endothelial cell count was between 1400 and 2000/mm^2^; all other transplants were optical transplants with a cell count above 2000/mm^2^ and a mean of 2237 cells/mm^2^. In the 4 grafts with the lower endothelial cell count, we observed one recurrent perforation. The Charlson Comorbidity Index at the time of surgery was not statistically significantly different between the two groups (*p* = 0.84) (Table 1).

**Table 1:** Baseline characteristics and causes for perforation of patients receiving AMT or PK

	AMT (*n* = 39)	PK (*n* = 39)	Significance
Mean age (years)	76 ± 16	72 ± 15	*p* = 0.2
Gender: female (*n* (%))	17 (44%)	19 (49%)	*p* = 0.8
Right eyes (*n* (%))	22 (46%)	23 (59%)	*p* = 1.0
Perforated area (% of the entire cornea)	0.8% ± 1.0%	1.2% ± 0.7%	*p* = 0.009
Mean follow-up (days)	460 ± 514	511 ± 526	*p* = 0.6
Charlson Comorbidity Index	1.8 ± 1.8	1.7 ± 1.7	*p* = 0.84

Cause (*n* in AMT (%)/*n* in PK (%)): bacterial (10 (26%)/6 (15%)), herpetic (2 (5%)/8 (21%)), exposure (4 (10%)/3 (8%)), neurotrophic (4 (10%)/7 (18%)), rheumatic (1 (3%) /2 (5%)), ocular rosacea (2 (5%)/1 (3%)), peripheral ulcerative keratitis (5 (13%)/0 (0%)), ocular cicatricial pemphigoid (2 (5%)/0 (0%)), iatrogenic (3 (8%)/2 (5%)), traumatic (2 (5%)/0 (0%)), bullous keratopathy (1 (3%)/0 (0%)), keratoconus (0 (0%)/1 (3%)), mycotic (0 (0%)/1 (3%)), atopic keratoconjunctivitis (0 (0%)/1 (3%)), Stevens-Johnson syndrome (0 (0%)/1 (3%)), acanthamoeba (0 (0%)/1 (3%)), uncertain cause (3 (8%)/5 (13%))

### Recurrence of corneal perforation

The rate of recurrence of the corneal perforation did not differ significantly between the two surgical approaches (*p* > 0.99). It occurred in 10 of 39 patients (26%) after AMT and in 9 of 39 patients (23%) after PK within the follow-up period. Time until recurrent perforation was also not significantly different between the two groups (AMT: 153 ± 158 days; PK: 133 ± 140; *p* = 0.97). Secondary surgeries in the AMT group were additional AMT in most cases, whereas 5 eyes in the AMT group required a definitive tectonic PK. One eye had to be enucleated due to failure of all previous surgical procedures. With PK as the primary treatment, 4 eyes with recurrent perforation required an additional AMT and 4 eyes a second tectonic PK. One enucleation had to be performed. The duration of the inpatient stay was statistically shorter in AMT, with 5 ± 3 days, compared with 11 ± 5 days for those receiving a PK (*p* < 0.001). The number of recurrent perforations sorted by the cause for perforation can be seen in Supplementary Material 1.

### Mortality and Charlson Comorbidity Index

During the study, we observed that 16 of 39 patients (41%) in the AMT group and 11 of 39 patients (28%) after PK died during the period of observation.

**Fig. 1:**
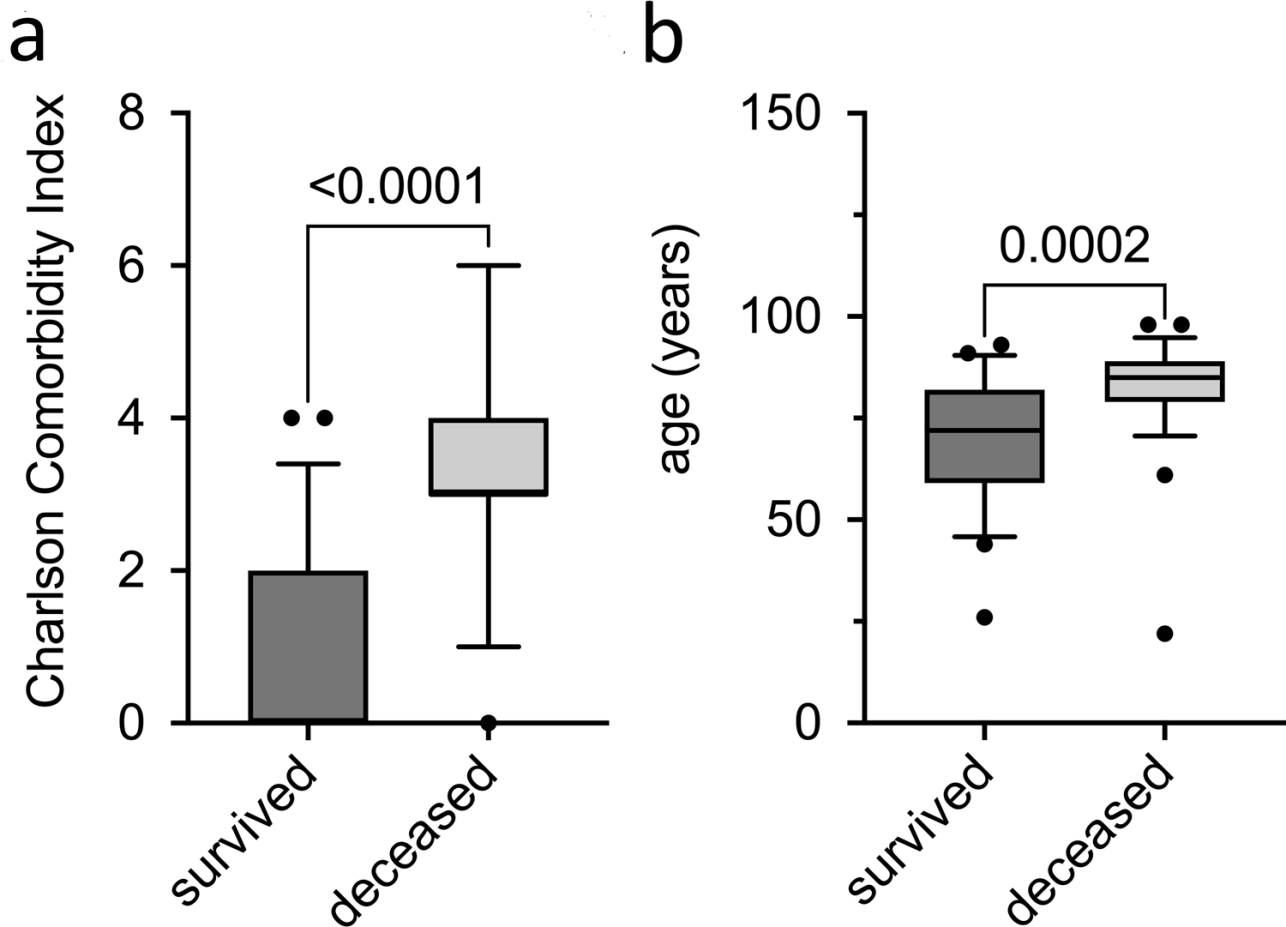
Both the Charlson Comorbidity Index (*p* < 0.0001) (**a**) and the age at the time of surgery (*p* = 0.0002) (**b**) are statistically significantly higher in those who died

The mortality rate was not statistically significant between both groups (*p* = 0.2). The Charlson Comorbidity Index was calculated based on the medical history known to us at the time of surgery, in order to figure out any possible reasons. The score was 0.95 ± 1.10 in those who did not die after surgery and 3.41 ± 1.53 in those who deceased during the study follow-up, reflecting a statistically significant difference (*p* < 0.0001) and suggesting a higher disease burden in those who died. In addition, the age was significantly higher (*p* = 0.0002) in those who deceased with 82 ± 14 years than in those who did not die with 71 ± 14 years (Fig. 1). Interestingly, among the patients who died, the Charlson Comorbidity Index was not statistically significantly different between AMT and PK (*p* = 0.09). To measure the effect of the potentially influencing factors patient age and Charlson Comorbidity Score on patient death, a Cox regression hazards model was calculated on the outcome measure death independent of surgical method. Per point in the Charlson Comorbidity Score, the risk of dying increases 1.8-fold (95% confidence interval: 1.4–2.3; *p* < 0.05) and per year of life 1.1-fold (95% confidence interval: 1.0–1.1; *p* < 0.05).

### Competing risk analysis

**Fig. 2:**
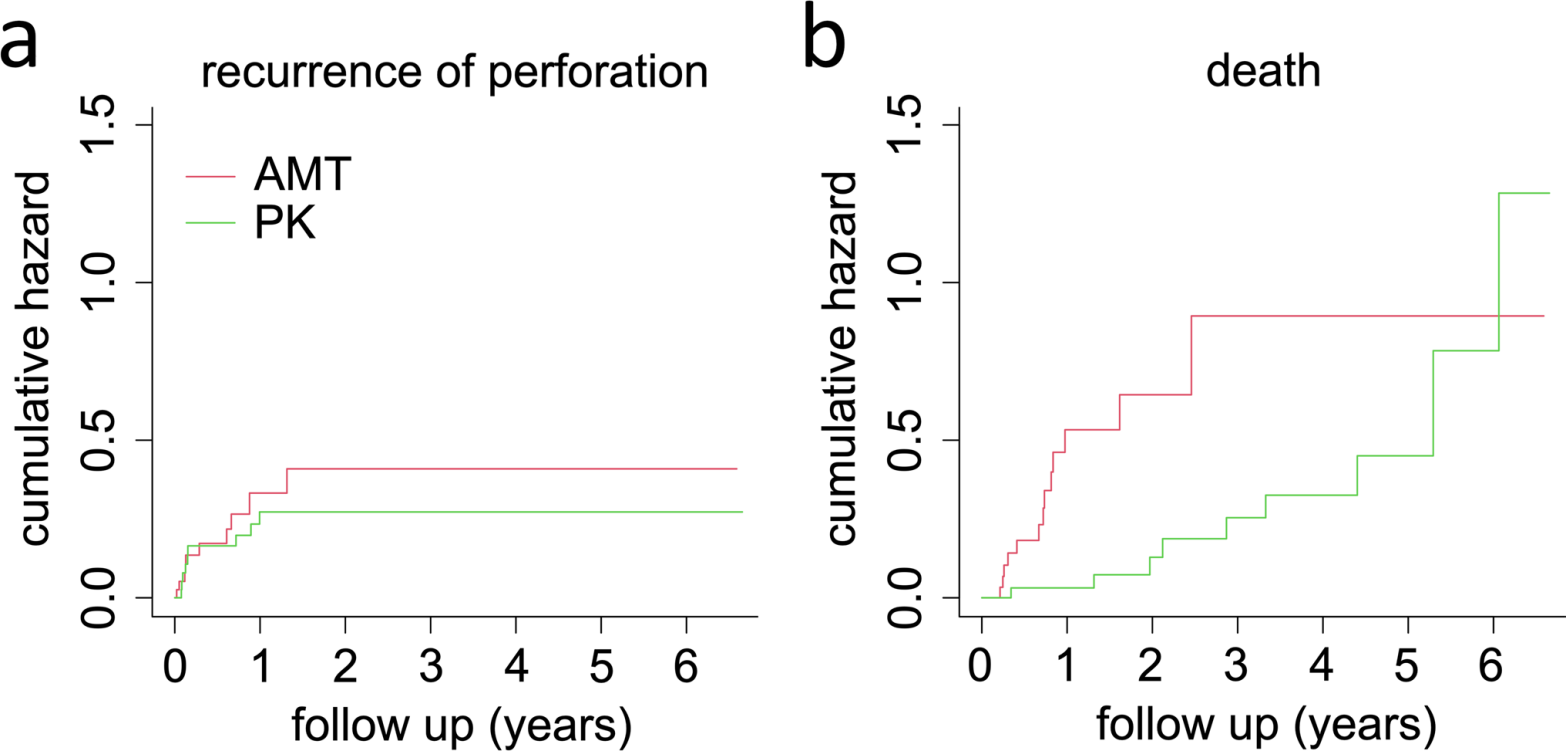
Corneal reperforation occurs equally in PK and AMT, whereas death occurred earlier in AMT. Depicted are the cumulative hazards for corneal recurrent perforation (**a**) and death (**b**) in both groups AMT and PK. The proportional hazards model of recurrent perforation after primary care was not significantly different (*p* = 0.5) between AMT and PK

Postoperatively, the cumulative hazard of corneal recurrent perforation initially increases sharply in both groups, with the highest risk within the first 2 years after surgery. Thereafter, it remains constant from about the second postoperative year. The cumulative hazard of death increases sharply in AMT up to three years postoperatively. In PK, it is initially low and increases only gradually. The Cox proportional hazards model was used to quantify the effects of AMT and PK on the risk of recurrence after adjustment of the influencing variables patient age and Charlson comorbidity score. The adjusted risk of corneal perforation recurrence is reduced by 0.7-fold (confidence interval: 0.3–1.8; *p* = 0.5) with PK compared to AMT (Fig. 2).

### Visual acuity at the last follow-up

As a secondary outcome, corrected visual acuity at the last follow-up was observed in both groups (Table 2). The distribution of visual acuity between the two categories AMT and PK did not vary statistically significantly (*p* = 0.7).

**Table 2:** A categorial distribution of corrected visual acuity (logMAR) receiving AMT or PK is shown. The visual acuity between the two categories AMT and PK did not vary statistically significantly at the last follow-up (*p* = 0.7)

Corrected visual acuity (log MAR)	AMT (number of patients)		PK (number of patients)	
	Initial presentation	Last follow-up	Initial presentation	Last follow-up
< 0.0–0.29	0	0	0	0
0.3–0.7	7	7	1	4
> 0.7	9	8	8	15
Counting fingers	3	4	3	2
Hand motion	12	11	14	10
Light perception	5	6	12	5
No light perception	1	0	0	0

## Discussion

This study compared the long-term success rates of the procedures AMT and PK for the treatment of corneal perforation. Both surgical methods show good tectonic results and no statistically significant difference regarding the recurrent perforation rate. In both groups, about a third of the patients died during the follow-up period. Therefore, the decision regarding the appropriate method for each patient should be based on a combination of multiple factors.

The rate of corneal perforation recurrence and failure of tectonic outcome observed in our study is approximately the same as in other studies. Failure rates determined by recurrent perforations range approximately from 0 to 30%, depending on the study, pathology, and follow-up time. In a series of 20 patients with various pathologies that resulted in perforations, including autoimmune and neurotrophic ulcers, a primary tectonic result was achieved in 80% after the first PK [12]. Slightly better results are obtained in series in which only perforated infectious ulcers were observed. In 43 patients, a tectonic success of 97.6% was achieved with PK [13]. These results are in strong contrast to a significantly worse outcome of PK for perforated bacterial ulcers, as determined by another group. The overall outcomes were clear graft in 50 (21.8%) eyes, failed graft in 139 (60.7%) eyes, evisceration in 19 (8.3%) eyes, phthisis bulbi in 14 (6.1%) eyes, recurrent PK in 2 (0.9%) eyes, and anterior staphyloma in 1 (0.4%) eye [14]. Approximately the same numbers are observed for amniotic membranes. In a larger series that also included several different causes, a failure rate of 33% was noted for the first AMT surgery [4]. This contrasts with a 100% success rate reported after a new technique using an AMT roll-in filling technique combined with multilayer amniotic membrane coverage [15].

Surgical technique had no effect on the primary outcome in our study. Also, the rate of recurrent perforation was on average not higher when grafts with lower endothelial cell count were used in comparison to the whole PK group. We hypothesize that the explanation for failure could be recurrence or persistence of the underlying disease. Unlike in bacterial ulcers or trauma, in which the underlying cause can often be eliminated, a second perforation could be due to persistent or recurrent disease that can be caused by various other pathologies [13]. Clinically, determination of the cause is of high interest because it mostly influences the treatment to prevent recurrence. However, the primary goal of ulcer therapy is to restore an intact globe, which is initially possible in almost all cases but has a failure rate [12]. It has been shown in other studies that visual acuity in ulcer patients is usually less than 0.2 logMAR after therapy [16]. Improving visual acuity is a secondary but important goal. In our study, both AMT and PK yielded equal results in visual acuity within the follow-up period.

Both techniques used in our study have certain advantages and disadvantages. AMT is widely used to treat external ocular diseases because of its anti-inflammatory, anti-infective, and anti-fibrotic effects and due to its good biocompatibility [17]. Because of this potential, AMT has beneficial biologic effects in most pathologies in addition to providing tectonic support. AMT also can be shaped into nearly every form, as it can be easily cut, folded, and sutured. The graft then integrates into the cornea and can restore tissue loss. It does not suffer from rejection as much as PK does and is a short-lasting procedure. In addition, it is often possible to avoid general anesthesia with AMT: In our study, 56% of the AMT was performed in topical or local anesthesia while all PK patients underwent surgery under general anesthesia. The duration of the inpatient stay was also statistically shorter in our AMT group compared to PK. Moreover, transplant availability in most regions is better for AMT. It is a good technique for smaller perforations and in cases of stroma loss. On the other hand, PK can treat both large and small perforations with extensive tissue necrosis and has some advantages regarding clear optic media but requires a long healing time and has a high risk of long-term transplant decompensation.

In our study, a high loss to follow-up was observed partly due to high mortality. This is comparable to what has been observed in literature. In another study on corneal perforations, 20% of patients died during the 36-month follow-up period: three patients due to end-stage cancer, two patients due to complications from chronic autoimmune disease, two patients due to end-stage renal disease and other complications from diabetes, and two patients due to chronic heart disease [18]. To look for an underlying reason for the deaths in our study, the pre-existing morbidity on the day of surgery was determined using the Charlson Comorbidity Index. The score is a validated and simple method for estimating the risk of death due to comorbidities and is widely used. We calculated a much higher index for the deceased at the time of surgery. The score for those who died was on average 3.41 ± 1.53. The 1-year mortality rates for the different scores were “0”: 12%, “1–2”: 26%, “3–4”: 52%, and “greater than or equal to 5”: 85%, respectively [10]. Those who died were on an average 11 years older than those who did not. Comorbidity and higher age as a general indicator of mortality [10] at the time of surgery might therefore be the likely explanation for the higher number of deaths occurring during the follow-up period. In addition, the corneal perforation might be a symptom of an underlying life-threatening systemic disease. This has been documented for vasculitis associated with peripheral ulcerative keratitis or necrotizing scleritis, where death has been linked to nonadministration of systemic immunosuppressants [19].

In addition to amniotic membranes, several other techniques have been described for the treatment of perforated corneal ulcers. All techniques have in common that they aim to seal the perforation and metabolically and mechanically support the healing of the cornea [20]. A PK requires the longest time to heal because the sutures are not removed for approximately one year. The decision to use a particular technique depends on the size, cause, location, and potential for visual recovery. A goal may also be to avoid emergency PK or to create suitable conditions for future optic PK [21]. In a larger percentage of patients, a single intervention is not enough, which is also supported by our data. Other techniques that have been described include sutureless tectonic pull-through mini DSAEK [22], transplantation of SMILE lenticules [23], or deep anterior lamellar keratoplasty [24]. More classic methods include tissue adhesives, such as cyanoacrylate glue [25], and conjunctival flap surgery which can also successfully restore ocular surface integrity [21]. Tenon patch grafts are also used in the treatment of corneal perforations [26]. In very rare cases, treatment may be unnecessary under close observation, such as when the perforation is very small, has no leakage, and is stable, e.g., after iris plug [27]. In our study with several heterogenous reasons for perforations, we achieved a success rate of 75% of patients treated with one procedure.

Our study encountered some limitations. Compared to the literature and considering the rarity of the disease, this study has a high number of cases. However, for the complex question, a higher case number would be useful. A multicentric design would be a possible option for future studies. The retrospective design and the heterogenous distribution of pathologies in the groups are two drawbacks. The groups were not fully comparable, as the perforations treated by PK were slightly but significantly larger than those in AMT. The retrospective design of the study prevents a prospective masked distribution of patients between the two surgical methods. Furthermore, the cause and size of the perforation play a role in the decision for a surgical method since larger perforations or perforations, e.g., caused by aggressive infectious pathogens, tend to be treated with PK rather than AMT. Future studies should focus on single pathologies and possibly be designed prospectively. All other factors were comparable, whereas a matching regarding the several possible underlying pathologies was not possible.

In conclusion, both surgical treatment methods showed good results, no statistically significant difference regarding recurrent perforation rate, and a markedly reduced visual acuity. In both groups, about a third of the patients died during the follow-up period, which might be explained by age and comorbidities at the time of surgery. Therefore, the decision regarding the appropriate method for each patient should be based on a combination of all underlying factors.

## Supplementary Information

Below is the link to the electronic supplementary material.

Supplementary file1 (DOCX 16 KB)

## References

[1] Stamate A-C, Tătaru CP, Zemba M (2019). Update on surgical management of corneal ulceration and perforation. Romanian J Ophthalmol.

[2] Deshmukh R, Stevenson LJ, Vajpayee R (2020). Management of corneal perforations: An update. Indian J Ophthalmol.

[3] Jhanji V, Young AL, Mehta JS (2011). Management of corneal perforation. Surv Ophthalmol.

[4] Krysik K, Dobrowolski D, Wylegala E, et al. Amniotic Membrane as a Main Component in Treatments Supporting Healing and Patch Grafts in Corneal Melting and Perforations. J Ophthalmol 2020; 2020: 4238919. 2020/03/10. 10.1155/2020/4238919. 10.1155/2020/4238919 PMC7042504 32148944

[5] Nurözler AB, Salvarli S, Budak K (2004). Results of therapeutic penetrating keratoplasty. Jpn J Ophthalmol.

[6] Lee S-H, Tseng SC (1997). Amniotic membrane transplantation for persistent epithelial defects with ulceration. Am J Ophthalmol.

[7] Jirsova K, Jones GL (2017). Amniotic membrane in ophthalmology: properties, preparation, storage and indications for grafting—a review. Cell Tissue Banking.

[8] Kjaergaard N, Hein M, Hyttel L (2001). Antibacterial properties of human amnion and chorion in vitro. Eur J Obstet Gynecol Reprod Biol.

[9] Schneider CA, Rasband WS, Eliceiri KW (2012). NIH Image to ImageJ: 25 years of image analysis. Nat Methods.

[10] Charlson ME, Pompei P, Ales KL (1987). A new method of classifying prognostic comorbidity in longitudinal studies: development and validation. J Chronic Dis.

[11] Quan H, Li B, Couris CM (2011). Updating and validating the Charlson comorbidity index and score for risk adjustment in hospital discharge abstracts using data from 6 countries. Am J Epidemiol.

[12] Hanada K, Igarashi S, Muramatsu O (2008). Therapeutic keratoplasty for corneal perforation: clinical results and complications. Cornea.

[13] Dogan C, Arslan OS (2019). Outcomes of Therapeutic and Tectonic Penetrating Keratoplasty in Eyes with Perforated Infectious Corneal Ulcer. Turk J Ophthalmol.

[14] Chatterjee S, Agrawal D (2020). Recurrence of Infection in Corneal Grafts After Therapeutic Penetrating Keratoplasty for Microbial Keratitis. Cornea.

[15] Fan J, Wang M and Zhong F (2016) Improvement of Amniotic Membrane Method for the Treatment of Corneal Perforation. BioMed research international 2016: 1693815. 2016/06/18. 10.1155/2016/1693815. 10.1155/2016/1693815 PMC4893576 27314009

[16] Yalniz-Akkaya Z, Burcu A, Doğan E (2015). Therapeutic penetrating keratoplasty for infectious and non-infectious corneal ulcers. Int Ophthalmol.

[17] Tseng SC (2001). Amniotic membrane transplantation for ocular surface reconstruction. Biosci Rep.

[18] Rush SW and Rush RB 2016 Outcomes of Infectious versus Sterile Perforated Corneal Ulcers after Therapeutic Penetrating Keratoplasty in the United States. J Ophthalmol 2016: 6284595. 2017/01/11. 10.1155/2016/6284595. 10.1155/2016/6284595 PMC5187482 28070416

[19] Ogra S, Sims JL, McGhee CNJ (2020). Ocular complications and mortality in peripheral ulcerative keratitis and necrotising scleritis: The role of systemic immunosuppression. Clin Exp Ophthalmol.

[20] Palioura S, Henry CR, Amescua G (2016). Role of steroids in the treatment of bacterial keratitis. Clin Ophthalmol (Auckland, NZ).

[21] Oostra TD, Mauger TF (2020). Conjunctival Flaps: A Case Series and Review of the Literature. Eye Contact lens.

[22] Roberts HW, Gunasekera CD, Law EM, et al. Sutureless Tectonic Mini-Descemet's Stripping Automated Endothelial Keratoplasty ("mini-DSAEK") for the management of corneal perforations. Eur J Ophthalmol 2021: 11206721211050034. 2021/10/19. 10.1177/11206721211050034. 10.1177/11206721211050034 34657450

[23] Yang H, Zhou Y, Zhao H (2020). Application of the SMILE-derived lenticule in therapeutic keratoplasty. Int Ophthalmol.

[24] Nguyen HT, Pham ND, Mai TQ (2021). Tectonic Deep Anterior Lamellar Keratoplasty to Treat Corneal Perforation and Descemetocele from Microbial Keratitis. Clin Ophthalmol (Auckland, NZ).

[25] Rodriguez EN, Townsend WM, Stiles J (2021). Double drape tectonic patch with cyanoacrylate glue for surgical repair of corneal defects: 8 cases. Vet Ophthalmol.

[26] Sharma N, Singhal D, Maharana PK (2019). Tuck-In Tenon Patch Graft in Corneal Perforation. Cornea.

[27] Muceniece L, Markevica I, Laganovska G (2020). Corneal Perforation Self-Healing with an Iris Plug in the Cornea. Case Rep Ophthalmol.

