# Supplementary material for: Recurrence of perforation and overall patient survival after penetrating keratoplasty versus amniotic membrane transplantation in corneal perforation
Source: Graefes Arch Clin Exp Ophthalmol. 2023 Jan 21;261(7):1933–40. doi: 10.1007/s00417-022-05914-0 (PMC10272250; doi:10.1007/s00417-022-05914-0)
Supplement: Supplementary file 1 — Supplementary file1 (DOCX 16 KB) [file 417_2022_5914_MOESM1_ESM.docx]

Table 3: Causes of perforations and number of recurrent perforations in those groups.

| Cause | AMT (n = 39) | | PK (n = 39) | |
| --- | --- | --- | --- | --- |
|  | **Number of listed causes** | **Number of recurrent perforations (overall number: 10)** | **Number of listed causes** | **Number of recurrent perforations (overall number: 9)** |
| bacterial | 10 (26%) | 3 | 6 (15%) | 1 |
| herpetic | 2 (5%) | 1 | 8 (21%) | 2 |
| exposure | 4 (10%) | 1 | 3 (8%) | 0 |
| neurotrophic | 4 (10%) | 0 | 7 (18%) | 3 |
| rheumatic | 1 (3%) | 0 | 2 (5%) | 1 |
| ocular rosacea | 2 (5%) | 0 | 1 (3%) | 0 |
| peripheral ulcerative keratitis | 5 (13%) | 2 | 0 (0%) | 0 |
| ocular cicatricial pemphigoid | 2 (5%) | 2 | 0 (0%) | 0 |
| iatrogenic | 3 (8%) | 1 | 2 (5%) | 1 |
| traumatic | 2 (5%) | 0 | 0 (0%) | 0 |
| bullous keratopathy | 1 (3%) | 0 | 0 (0%) | 0 |
| keratoconus | 0 (0%) | 0 | 1 (3%) | 0 |
| mycotic | 0 (0%) | 0 | 1 (3%) | 0 |
| atopic keratoconjunctivitis | 0 (0%) | 0 | 1 (3%) | 0 |
| Stevens-Johnson Syndrome | 0 (0%) | 0 | 1 (3%) | 0 |
| acanthamoeba | 0 (0%) | 0 | 1 (3%) | 0 |
| uncertain cause | 3 (8%) | 0 | 5 (13%) | 1 |
